# Creating a prediction model for invasive candidiasis in the intensive care unit using a case control design: a European multicentre approach

**DOI:** 10.1186/s12879-025-10644-9

**Published:** 2025-05-04

**Authors:** P. M. B. Benders, J. Schouten, A. Vena, J. B. Buil, E. Bronkhorst, M. Bassetti

**Affiliations:** 1 Department of Anesthesiology, Radboudumc, Nijmegen, The Netherlands; 2 Department of Intensive Care Medicine, Radboudumc, Nijmegen, The Netherlands; 3 Department of Infectious Diseases, San Martino University Hospital, Genua, Italy; 4 Department of Microbiology, Radboudumc, Nijmegen, The Netherlands; 5 Department of Biostatistics, Radboudumc, Nijmegen, The Netherlands

**Keywords:** Candidaemia, Candidiasis, ICU, Prediction model, LASSO

## Abstract

**Purpose:**

Invasive candidiasis (IC) has a high attributable morbidity and mortality in patients in the intensive care unit (ICU). Current diagnostic tools lack sensitivity, introduce delay or have not been validated for regular use. As early treatment has proven vital for survival, multiple prediction models have been proposed but have not been validated for multinational implementation. In this study we propose to find factors predisposing the ICU patient to develop IC. We hope to develop an alternative prediction model using a large international dataset.

**Methods:**

Using ICU-acquired IC as primary endpoint we retrieved retrospective information about 285 cases and 285 matched controls from the EUCANDICU database. Data about comorbidities, severity of illness and known risk factors for IC were available. We identified 31 independent risk factors using univariate analysis. A random subset of 80% of the observations were used to find the optimal prediction model. The selection of predictors was done using the LASSO technique, using λ = 1SE as regularization parameter. This choice for λ implies that a small amount of precision of the prediction is sacrificed to improve the external validity. The remaining 20% of cases were used to assess the predictive performance of the model.

**Results:**

Among other factors SAPS II score, SOFA score, past infection, renal impairment and the presence of multiple *Candida* colonization sites were all independently associated with an increased risk of developing IC. We incorporated 22 of 31 variables in a LASSO regression analysis which showed an AUROC of 0.7433.

**Conclusion:**

Predicting which ICU patient will develop invasive candidiasis remains challenging, despite using an alternative methodology in a large multinational database. The performance of this prediction model is not good enough to be used in clinical practice.

**Supplementary Information:**

The online version contains supplementary material available at 10.1186/s12879-025-10644-9.

## Introduction

The incidence of invasive fungal infections in the ICU department has increased over the past decades [1]. The majority of these infections (80%) is caused by *Candida* species [1, 2].

The limited available data on ICU-acquired invasive candidiasis (IC) suggest that candidemia and invasive abdominal candidiasis (IAC) account for most of ICU-acquired IC [3–5], with IAC being more common than previously recognized [6]. Candidemia is considered the fourth most common bloodstream infection in the ICU [7]. ICU-acquired IC (comprising both IAC and candidemia) is associated with a significant increase in mortality, morbidity, health care costs and a prolonged length of ICU stay [8, 9]. With early detection and treatment improving survival [10], the need for a quick and reliable diagnostic tool for ICU-acquired IC is evident. The current gold standard for detecting IC is direct detection through cultures. Alas, the sensitivity of blood cultures is far from ideal, with a sensitivity of 21–71% reported in autopsy studies [6]. Indirect testing with surrogate markers have been studied broadly, but their reported clinical relevance is controversial [11–14]. Polymerase-chain-reaction (PCR) assays are subject of extensive research and some have produced promising results [15] however, limited validation and standardization have slowed their clinical implication [16] and to this date there is no consensus about the utilization of PCR.

Contemplating these challenges in diagnosing and treating ICU-acquired IC it is paramount that early intervention strategies are implemented in current practice to prevent these problems from growing. A prediction model can prove a valuable tool as part of such an intervention strategy. Various prediction models have previously been suggested but reported specificity (and thereby practical usefulness) is disappointing (51–81%) [17, 18]. Furthermore, the published prediction models are only applicable in the country where they have been proposed. Validation of these models in other countries has proven unsuccessful: [19] most likely different countries deal with different *Candida* species, resistance patterns and choose a different approach in antifungal prophylaxis in ICU patients [2].

Using a large European dataset collected in the EUCANDICU project [20], we were able to analyze clinical data from patients with ICU-acquired IC in a multinational setting. Our objective was to determine the significance of these predictive factors for IC in this large ICU population. Using these factors, we aimed to create a practical prediction model to determine patients at risk for ICU-acquired IC who could potentially benefit from early antifungal prophylaxis or treatment.

## Methods

Several reviews have evaluated the quality of published reports that describe the development or validation prediction models. Reporting was generally found to be poor [21–24]. Therefore we used the TRIPOD checklist to compose this paper [25].

### Study design

This study is a case control study where data were retrospectively collected from the participating hospital’s respective patient records.

### Source of data

This study was performed using the data collected for the EUCANDICU study [20]. The EUCANDICU study is a multicenter, international, retrospective case–control study of invasive *Candida* infections in ICU patients admitted between January 2015 and December 2016, initiated by the European Society of Clinical Microbiology and Infectious Diseases (ESCMID).

### Participants

The data were collected from 23 intensive care units (ICUs) in 22 large tertiary care hospitals in Europe:9 in Italy (111 patients), 4 in France (43 patients), 2 in Greece (8 patients), 1 in Belgium (17 patients), 1 in Czech Republic (19 patients), 1 in Germany (47 patients), 1 in Ireland (5 patients), 1 in Portugal (8 patients), 1 in Spain (8 patients), and 1 in the Netherlands (19 patients). All patients who developed an episode of candidemia or a microbiologically documented IAC [26] during their stay in the ICU (at least 48 h after admission) were included in the study. ICU-acquired IC was defined as candidemia or IAC with signs and symptoms of infection developing at least 48 h after ICU admission. Candidemia and IAC were defined according to previously published definitions [26, 27]. More in detail, candidemia was defined as the presence of at least one positive blood culture for *Candida* spp. in patients with signs and symptoms of infection. IAC was defined as the presence of at least one of the following: (i) *Candida* detection by direct microscopy or growth in culture from necrotic or purulent intra-abdominal specimens obtained by percutaneous aspiration or during surgery; (ii) growth of *Candida* from the bile or intra-biliary duct devices, plus biopsy of intra-abdominal organs; (iii) growth of *Candida* from blood cultures in the presence of secondary or tertiary peritonitis in the presence of no other pathogens; (iv) growth of *Candida* from drainage tubes inserted less than 24 h before culture sampling [26]. All patients suitable for inclusion were identified starting from the microbiological laboratory databases of the participating hospitals, and subsequent review of clinical records.

The controls were matched according to the following criteria: 1) 18 years and older, and 2) admitted to the same ICU as the case, and 3) ICU stay longer than or equal to the ICU stay before manifestation of IC in the case. We searched for eligible controls with an admission date as close as possible to the case’s admission date. If more than one eligible control was found we randomly selected one of them. The moment of enrollment for cases was defined as the moment of the positive sample collection. Controls were enrolled at the day when equal duration of ICU stay to enrollment was reached as the matched case.

### Outcome

The primary endpoint in this study was ICU-acquired IC as documented in the previous subheading. No blinding took place as this concerns retrospectively collected data.

### Sample size

In this study we included 285 cases of ICU-acquired IC and we selected 285 paired controls. As data were retrospectively collected, no power calculation was made. All eligible patients were included in a period of two years.

### Predictors

We selected possible variables on their likelihood to influence occurrence of IC, based on previously published risk factors [2, 28–30]. Data concerning demographics, comorbidities, severity of illness and known risk factors during hospital and ICU stay were collected from the database.

The following comorbidities were assessed: diabetes mellitus (DM), chronic obstructive pulmonary disease, any infection in past three months, solid tumor, hematologic malignancy, human immunodeficiency virus, transplant, cirrhosis, kidney failure (eGFR < 60 ml/min), history of renal failure requiring dialysis, renal replacement therapy at the time of IC and burns. Also, at the moment of study enrollment we calculated the age-adjusted Charlson comorbidity index a 17-variable score that was validated for predicting comorbidity-attributable 10 year mortality [31].

Severity of illness was scored using the sequential organ failure assessment (SOFA) score [32] and simplified acute physiology score (SAPS II) [33] at ICU admission.

Previously described known risk factors include *Candida* colonization on enrollment (pharyngeal, respiratory tract, urinary tract, skin, wound or drainage) of 1 site or 2 or more sites, the number of surgical abdominal interventions up until the moment of enrollment, leakage or anastomosis, presence of a vascular device on enrollment, total parenteral nutrition (TPN) on enrollment, mechanical ventilation on enrollment, history of solid organ transplant and exposure to antibiotics (≥ 7 days) or antifungals (divided into echinocandins, azoles or amphotericin B) or immunosuppressive medications (divided in steroids and other) in the 30 days prior to enrollment.

### Missing data

We included less cases than in the original EUCANDICU study [20] because of in some controls for these cases, important variables were missing (e.g. colonization sites) so that these could not be adequately matched. We therefore decided to omit several participants. For controls, no data was available for sepsis or septic shock at time of inclusion. Therefore, these variables could not be used in final analyses.

### Statistical analysis/methods

We compared cases and controls for demographic and clinical characteristics. Because of our extensive sample size, we assumed normality for the means of the continuous data in our patient groups. For categorical (dichotomous) data we used the Chi-squared test. Continuous data were tested by applying the unpaired t-test.

Too many potential predictors were selected to fit within a potential easily applicable prediction model. Therefore, a procedure for predictor selection was needed. To avoid filter (correlation based) and wrapper (e.g. forward and backward selection) methods, parameter selection was performed using a least absolute shrinkage and selection operator (LASSO) algorithm [34]. A LASSO algorithm seeks to balance prediction errors and generalizability by using a penalty parameter lambda, which regulates to what extent the sum of betas is limited. In this process some of the beta coefficients will shrink to zero. This reduces variance and overfitting, and hence increase generalizability. This is especially interesting for our case with many predictors and (potentially) limited cases. Discriminative power of the model is measured by the area under the receiver operating characteristic (AUROC). An optimal LASSO model yields a value for the linear predictor for every patient. That can only be converted to an actual prediction if a cutoff is applied. But this cutoff is not part of the model. And it is conceivable that the same prediction model has different cutoffs in for instance different patient groups or different countries. To demonstrate the performance of the model, a plot is presented which shows the resulting sensitivities and specificities for a wide range of values for the cutoff. To this plot the values of the positive and negative predictive values for all cutoffs are added.

All statistical analyses were carried out using IBM SPSS Statistics 25, except the LASSO which was performed by the glmnet package, version 4.0–2 within R version 3.6.2

### Development/validation

We validated the model on the same dataset by running 80% of the cases versus 20% of cases to check for accuracy and applicability.

### Application of previously published Candida prediction models

For comparison of the performance of our developed *Candida* prediction models with previously published prediction models, we applied three previously prediction models to our dataset and calculated AUROC. The prediction rules were developed by Ostrosky et al. [35]. Rule 1 was defined by presence of the following risk factors: antibiotic use AND presence of CVC. Rule 2 was defined by: antibiotic use AND presence of CVC AND either surgery, immunosuppressive use, pancreatitis, TPN, dialysis or steroid use. Rule 3 by: antibiotic use OR presence of CVC AND at least two additional risk factors (surgery, immunosuppressive use, pancreatitis, TPN, dialysis or steroid use).

## Results

### Participants

The presented cohort consisted of 570 patients (*n* = 285 cases and *n* = 285 paired controls). In Table 1 all relevant demographic variables and potential risk factors for the development of IC and the distribution between cases and controls are shown. Univariate analysis showed significant differences in length of ICU stay (39,73 vs 26,60 days) and severity of illness scores. Other significant differences were seen for past infection, kidney failure, history of dialytic renal failure, renal replacement therapy, *Candida* colonization of more than one site, number of abdominal interventions, abdominal leakage or presence of anastomosis, TPN, recent exposure to antibiotics or to azoles.

**Table 1:** Demographics

**Demographics**	**Overall (mean + SD)**	**IC-group**	**Controls**	***p*-value**
**Gender (male)**	332 (58.9%)	165 (59.3%)	167 (58.6%)	0.865
**Age (mean ± SD)**	64.09 ± 13.945	63.54 ± 14.258	64.64 ± 13.628	0.348
**Length of stay**				
**ICU stay (days)**	33.17 ± 31.13	39.73 ± 35.74	26.60 ± 24.02	** < 0.001**
**Scores**				
**SAPS II at study enrollment**	41.41 ± 19.947	46.32 ± 20.174	36.49 ± 18.507	** < 0.001**
**SOFA at ICU admission**	7.11 ± 4.419	7.62 ± 4.461	6.59 ± 4.323	**0.005**
**Charlson at ICU admission**	5.6 ± 3.01	5.61 ± 3.04	5.60 ± 2.99	0.956
**Comorbidities**				
**Diabetes Mellitus**	135 (23.7%)	62 (21.8%)	73 (25.6%)	0.278
** COPD**	97 (17.0%)	42 (14.8%)	55 (19.2%)	0.147
**Past infection (3 months)**	234 (41.1%)	154 (54.0%)	80 (28.0%)	** < 0.001**
**Solid tumor**	165 (28.9%)	79 (27.8%)	86 (30.2%)	0.518
**Hematologic malignancy**	22 (3.9%)	15 (5.2%)	7 (2.4%)	0.082
**HIV**	9 (1.6%)	6 (2.2%)	3 (1%)	0.313
**Cirrhosis**	34 (6%)	22 (7.8%)	12 (4.2%)	0.077
**Kidney failure**	245 (43%)	144 (50.6%)	101 (35.4%)	** < 0.001**
**History of dialytic renal failure**	66 (11.6%)	48 (16.8%)	18 (6.4%)	** < 0.001**
**Renal replacement therapy**	119 (20.9%)	81 (28.4%)	38 (13.4%)	** < 0.001**
**Burns**	0 (0%)	0 (0%)	0 (0%)	-
**Known risk factors**				
***Candida* colonization 1 site**	135 (23.7%)	77 (27.0%)	58 (20.4%)	0.061
***Candida* colonization (2 ≥ sties)**	97 (17.0%)	65 (22.8%)	32 (11.2%)	** < 0.001**
**Number of surgical abdominal interventions**	1.1 ± 2.005	1.52 ± 2.32	0.68 ± 1.522	** < 0.001**
**Leakage of anastomosis**	76 (13.3%)	54 (19%)	22 (7.8%)	** < 0.001**
**Vascular device present**	529 (92.8%)	270 (94.8%)	259 (90.8%)	0.075
** TPN**	297 (52.1%)	174 (61.0%)	123 (43.2%)	** < 0.001**
**Mechanical ventilation**	438 (76.8%)	227 (79.6%)	211 (74%)	0.112
**Solid organ transplant**	23 (4%)	16 (5.6%)	7 (2.4%)	0.055
**Recent exposure to antibiotics**	372 (65.3%)	215 (75.4%)	157 (55%)	** < 0.001**
**Recent exposure to echinocandins**	65 (11.4%)	38 (13.4%)	27 (9.4%)	0.147
**Recent exposure to azoles**	62 (10.9%)	39 (13.6%)	23 (8%)	**0.031**
**Recent exposure to amphotericin B**	6 (1.1%)	3 (1.1%)	3 (1.1%)	1
**Recent exposure to steroids**	73 (13.0%)	40 (14.10%)	33 (11.6%)	0.319
**Recent exposure to other immunosuppressive drugs**	49 (8.6%)	31 (10.8%)	18 (6.4%)	0.052

Table depicting the demographics and the prevalence of potential risk factors for developing ICU-acquired IC for our study group and our control group. Univariate analysis was performed using the Chi-squared test for categorical (dichotomous) data. Continuous data were tested by applying the unpaired t-test

### LASSO regression

**Fig. 1:**
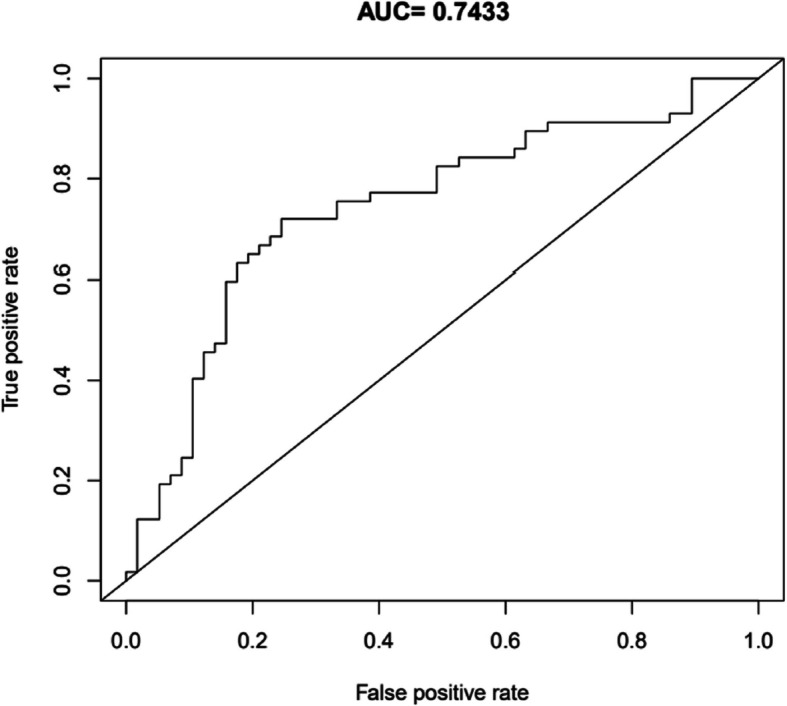
Prediction based model. Graph depicting the AUROC of the prediction model showing a mean AUROC of 0,7433 representing moderate to fair discriminative performance

**Fig. 2:**
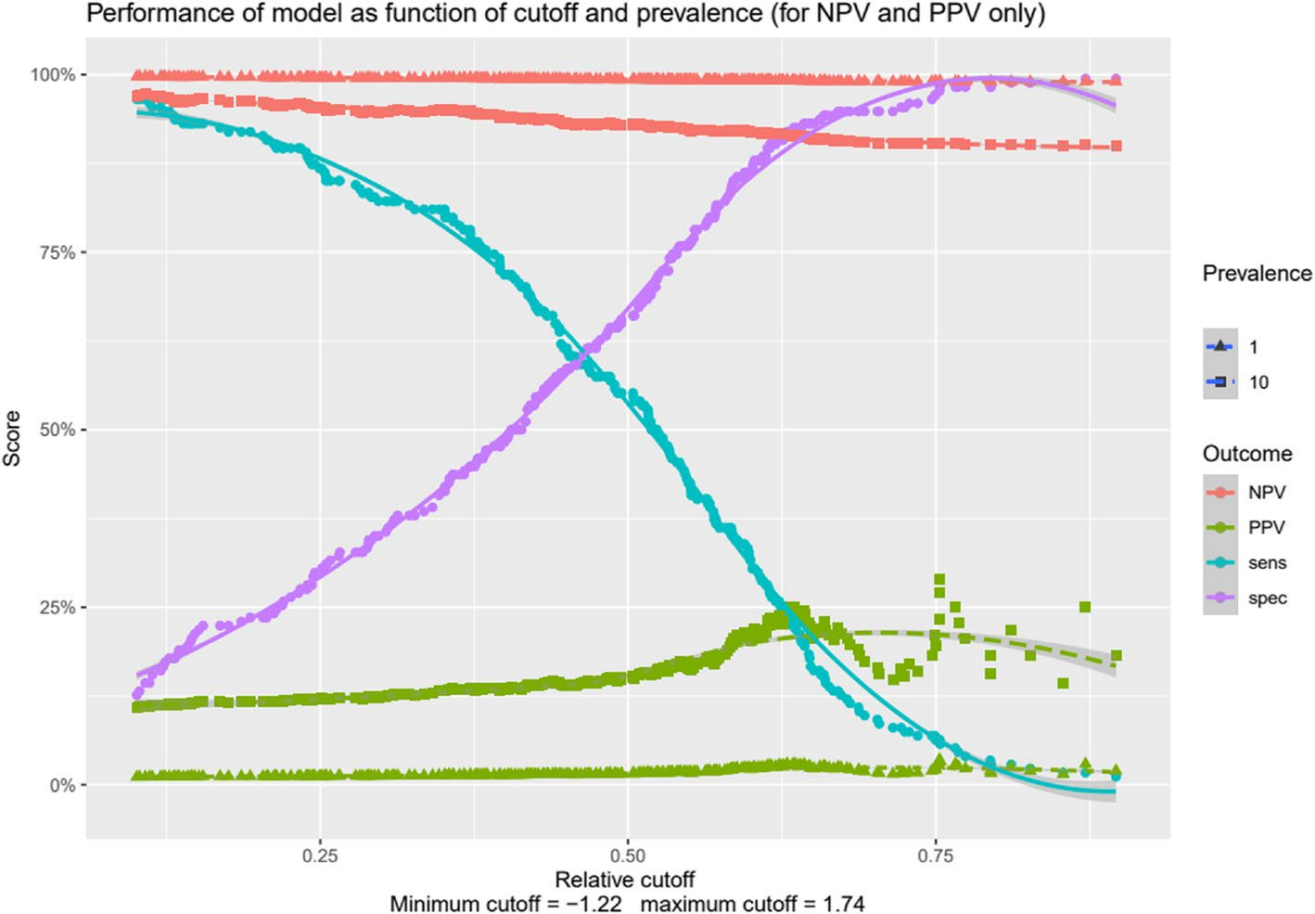
Sensitivity, specificity, positive and negative predictive values. Figure depicting sensitivity (light blue line) and specificity (purple line) for different cutoff points of the prediction model (the higher the cutoff, the more potential predictors the patient has). The negative predictive value (NPV) is shown in red in 2 lines (lines representing the NPV for a prevalence of 1% and 10%). The positive predictive value (PPV) is shown in green lines for the same prevalences

In Fig. 2 the resulting model from the LASSO procedure is presented. This model showed an AUROC of 0.7433 in the 20% of observations that were used to test the model performance. The ROC-curve is shown in Fig. 1. The individual conversion factors that LASSO assigned to each variable are presented in the appendix as supplementary material.

For a wide range of cutoffs the sensitivity/specificity combinations are shown in Fig. 2. A higher cutoff represents a patient with a higher number of potential risk factors as determined by the LASSO regression and vice versa. For theoretical prevalences of 1% and 10% of ICU acquired IC, corresponding positive and negative predictive values are shown.

### Previously published Candida prediction models

By applying the prediction rules defined by Ostrosky et al. [35] to our data, we found respective AUROCs of 0.6088 [0.5699–0.6477], 0.6351 [0.5957–0.6745] and 0.5842 [0.5448–0.6236] for rule 1, 2 and 3 respectively.

## Discussion

We aimed to develop a prediction model for IC based on the EUCANDICU database. LASSO regression analysis revealed an AUROC of 0,7433 for our prediction model which is considered to be of moderate to fair discriminative performance [36]. We show that for a higher cutoff (representing a patient with a higher number of potential risk factors as determined by the LASSO regression) specificity reaches a value close to 1 relatively quickly. The sensitivity is depicted as a mirrored sigmoid curve showing high values for a low cutoff point and low values for a high cutoff point. Sensitivity and specificity never reach high levels simultaneously and thus a well performing cutoff could not be defined. More importantly, the positive predictive value remains very low, irrespective of the level of theoretical prevalence (1 or 10%) of ICU acquired IC. These findings render the clinical usefulness of this model low. Thus, in our opinion, the combination of the sensitivity/specificity and low PPV is too weak to suggest a patient category for prophylactic or targeted treatment based on this predictive model. Because of this, no decision curve analysis was presented. Because we used internal validation of our data, we did not present calibration.

So, even though, to our knowledge, the EUCANDICU is the largest known dataset of patients with IC in the ICU, we were not able to present a methodologically robust prediction model. This is in line with previous studies that presented prediction models showed only poor to fair specificity [17, 37] and were not applicable to ICU settings from different countries [19].

Several other studies identified risk factors for the development of IC in ICU patients and developed predictive models for the prediction of IC in the ICU [17, 28, 35, 37, 38]. When comparing the previously published data about potential predictive factors for ICU-acquired IC to our data, our findings are found to be quite similar. For example, a recent, French study conducted by Poissy et al. [37] confirmed several well established risk factors for developing candidemia for critically ill patients such as total parenteral nutrition, septic shock and renal replacement therapy. The AUROC of their study is comparable to our study (0,768 vs 0,743). A short overview of predictive factors from the literature is shown in Table 2.

**Table 2:** An overview of known risk factors in literature

**Studies**	**Risk factors**
Poissy et al. [37]	2, 3, 6, 7, 8, 9
Leon et al. [17]	1, 3, 4, 5, 6
Ostrosky et al. [35]	3, 5, 6, 7
Shahin et al. [38]	1, 2, 4, 5, 9
Lortholary et al. [28]	2, 4, 5, 8

1 = Severity of illness (SOFA/SAPSII/APACHE)

2 = Central venous catheter

3 = (History of) Kidney failure or RRT

4 = *Candida* colonization

5 = Surgical abdominal interventions

6 = Total parenteral nutrition

7 = Recent use of antibiotics

8 = Recent use of antimycotics

9 = Previous infection, sepsis or septic shock

We sought to apply the previously developed prediction models to our data to see if the performance was comparable to our model.

Ostrosky studied three prediction rules for the identification of patients at risk for IC [35]; Rule 3 was identified as the most promising prediction rule including 34% of patients with candidemia with a specificity of 90%. The AUROC of these prediction rules (0.5842 to 0.6351) were slightly lower that the AUROC of our analysis (0.7433) and therefore these prediction rules have a low clinical usefulness when applied to our data.

We tried to run prediction models presented by Léon and Poissy et al. to our data. These scores could not be applied because information about severe sepsis, presence of previous septic shock and aminoglycoside use were not available in the EUCANDICU database.

### Limitations

The multinational aspect of our study presents us with the first possible limitation as different countries deal with diagnosis, prevention and treatment of fungal infections differently. For example, in the Netherlands, selective digestive decontamination (SDD, containing non-absorbable Amphotericin B oral paste and enteral solution) is routinely applied in all patients with an expected ICU stay > 48 h. This results in decolonization of *Candida* species in the digestive system within the first week of admission [39]. In other countries, other strategies may apply. This heterogeneity might increase the risk of bias: studies on a more homogenous population might be needed to validate a predictive algorithm specific to every country. However, the only effective way of retrieving sufficient data on patients with ICU-acquired IC is to collect the data internationally as invasive candidiasis has such a low incidence (2–14 cases per 100.000 (3)).

As this study was set up in a retrospective manner, it is inherently prone to (recall or misclassification) bias. We sought to prevent convenience sampling of controls by taking controls sharing most characteristics with our cases (without the primary endpoint of course). Nonetheless, as this selection was done retrospectively, it remains prone to some degree of selection bias as well as a degree of underreporting of variables relevant to developing ICU acquired IC. Also, the number of controls per case (1:1) is rather low for testing the data of cases. Ideally, more controls would have been included. Also, controls were not matched according to severity of illness. In practice, a decision tool would ideally help a clinician to decide to treat assumed IC in the most severely ill patients. This choice would be less relevant if a patient did not present with a septic profile or severely ill. The present matching of controls does not account for that.

One could argue that clinical applicability of the prediction model would have posed some issues. Namely using clinical scores (SAPS II, SOFA) within another prediction score is not practical. However, we sought to include mainly variables which were already proven to be predictive for development of IC.

Underestimation of positive predictive value (PPV) and overestimation of the negative predictive value (NPV) might occur because no patients with high suspicion of IC without positive blood cultures were included, keeping in mind that sensitivity of a positive blood culture (gold standard) is around 50% [6]. Unfortunately, in our dataset, some variables in the control population were not collected. These variables include septic shock and aminoglycoside use and blood transfusion [40], earlier recognized by other groups as predictors for developing IC.

### Interpretation

We sought to develop a generally applicable prediction model for the risk of IC in the ICU by using a regression analysis (LASSO) that, to our knowledge, is unique in this setting. By applying LASSO, the authors believe to have used the most fitting methodological regression analysis for this diverse, multinational population. Furthermore, we used 20% of our total cohort as a “validation cohort (training data)” to mitigate the potential distorting effect of the heterogeneity of our multinational population.

In our study, the specificity, sensitivity and PPV confirm that invasive candidiasis in the ICU patient is still incredibly difficult to predict accurately. This is most likely due to the low prevalence (< 1% in ICU patients [7]) of invasive candidiasis in combination with the heterogenous way the patient prone to developing invasive candidiasis clinically presents. Unfortunately, we believe that -at this time- there is not sufficiently robust data to support any prediction model for the development of IC in the ICU. The predictive factors that we found are in accordance with previous studies.

In contrast to using a prediction model, several studies have analyzed the performance of surrogate biomarkers for the identification of IC in ICU patients. A meta-analysis showed a combined sensitivity of 81% (95% CI 74 to 86%) [41] but studies show large heterogenicity [42]. Sensitivity and specificity may increase by combining BDG with CRP [43]. Others have studied a *Candida* IgG test for the identification of candidemia [44]. While these surrogate markers still have suboptimal performance for the identification of IC in ICU patients as single tests, including the results of these markers in predictive models could potentially improve the performance of predictive models resulting in a clinically applicable model to sensitively identify patients with IC [45, 46].

Because predicting which patients will develop invasive candidiasis remains incredibly difficult, early diagnostic efforts should be made in these suspect cases.

## Conclusion

Predicting which ICU patient will develop invasive candidiasis remains challenging, despite using an alternative methodology in a large multinational database. The performance of this prediction model is not good enough to be used in clinical practice.

## Take home message

It remains incredibly difficult to predict which patients will develop invasive candidiasis in the ICU department. There is not enough data at this time to suggest any clinically useful prophylactic intervention. Therefore, early diagnosis with subsequent early treatment is still a clinical case-by-case decision and of utmost importance.

## Supplementary Information

Supplementary Material 1.

## Data Availability

The datasets used and/or analysed during the current study are available from the corresponding author on reasonable request.

## Abbreviations

AUC: Area Under the Curve
AUROC: Area Under the Receiver Operating Characteristic
COPD: Chronic Obstructive Pulmonary Disease
DM: Diabetes Mellitus
ESCMID: European Society of Clinical Microbiology and Infectious Diseases
HIV: Human Immunodeficiency Virus
IAC: Invasive Abdominal Candidiasi
IC: Invasisve candidiasis
ICU: Intensive Care Unit
LASSO: Least Absolute Shrinkage and Selection Operator
NPV: Negative predictive value
PCR: Polymerase Chain Reaction
PPV: Positive predictive value
SAPS: Simplified Acute Physiology Score
SD: Standard Deviation
Sens: Sensitivity
Spec: Specificity
SOFA: Sequential Organ Failure Assessment
Spp: Species
TPN: Total Parenteral Nutrition
