# Supplementary material for: Creating a prediction model for invasive candidiasis in the intensive care unit using a case control design: a European multicentre approach
Source: BMC Infect Dis. 2025 May 4;25:655. doi: 10.1186/s12879-025-10644-9 (PMC12051287; doi:10.1186/s12879-025-10644-9)
Supplement: Supplementary file 1 — Supplementary Material 1. [file 12879_2025_10644_MOESM1_ESM.docx]

**Appendix**

**LASSO outcomes**

(Optimal model, as determined by the LASSO procedure. λ=1SE)

Individual scores for every variable in prediction model

**Predictor Beta**

Age -0,0086

SAPSII score 0,0275

SOFA score

Charlson score -0,0290

HIV

Solid tumor -0,0386

Dialitic renal failure 0,5779

COPD -0,1613

Burns

Diabetes -0,1181

Mechanical ventilation

Solid organ transplant

Cirrhosis

Previous infection 0,6971

Hematological malignancy 0,4902

Renal failure

Renal replacement therapy 0,1115

Number of abdominal surgical interventions 0,0676

Vascular device -0,2588

Abdominal device -0,1626

Parenteral nutrition 0,1807

Recent antibiotics 0,3628

Echinocandins -0,3516

Azoles

Amphotericin B -0,1412

Steroid treatment

Other immunosuppressants

Previous abdominal surgery

Anastomotic leakage 0,6321

Vascular device removal 0,5122

Candida colonization 1 site 0,1872

Candida colonization 2+ sites 0,6357

**List of abbreviations**

AUC = Area Under the Curve

AUROC = Area Under the Receiver Operating Characteristic

COPD = Chronic Obstructive Pulmonary Disease

DM = Diabetes Mellitus

ESCMID = European Society of Clinical Microbiology and Infectious Diseases

HIV = Human Immunodeficiency Virus

IAC = Invasive Abdominal Candidiasi

IC = Invasisve candidiasis

ICU = Intensive Care Unit

LASSO = Least Absolute Shrinkage and Selection Operator

NPV = Negative predictive value

PCR = Polymerase Chain Reaction

PPV = Positive predictive value

SAPS = Simplified Acute Physiology Score

SD = Standard Deviation

Sens = Sensitivity

Spec = Specificity

SOFA = Sequential Organ Failure Assessment

Spp = Species

TPN = Total Parenteral Nutrition
